# Wastewater treatment plant effluent alters particle-associated bacterial assemblages in an intermittent stream

**DOI:** 10.3389/fmicb.2026.1880555

**Published:** 2026-08-11

**Authors:** John J. Kelly, Karl Gaisser, Jennifer D. Drummond, José Gonçalves, Susana Bernal, Eugènia Martí

**Affiliations:** 1 Department of Biology, Loyola University Chicago, Chicago, IL, United States; 2 School of Geography, Earth and Environmental Science, University of Birmingham, Birmingham, United Kingdom; 3 MARE—Marine and Environmental Sciences Centre, ARNET—Aquatic Research Network, NOVA School of Science and Technology, NOVA University Lisbon, Campusda Caparica, Caparica, Portugal; 4 Integrative Freshwater Ecology Group, Centre of Advanced Studies of Blanes (CEAB-CSIC), Blanes, Spain

**Keywords:** bacteria, benthic, community composition, community function, fine particulate matter, intermittent stream, wastewater treat plants (WWTP), wastewater treatment effluent

## Abstract

Wastewater Treatment Plants (WWTPs) can be point-sources of nutrients, organic matter, anthropogenic contaminants, and microbes to lotic environments, and the input of WWTP effluent can alter the activity and composition of stream microbial assemblages in the benthos and water column. Within the stream benthos, fine particulate matter (FPM) supports especially high levels of microbial activity, but the effect of WWTP effluents on the microbial communities specifically associated with benthic FPM in rivers has not been studied. The present work sought to address this knowledge gap by analyzing FPM quantity, nutrient content, microbial activity, and bacterial assemblage composition in FPM samples collected from sites upstream and downstream of a WWTP effluent input into an intermittent Mediterranean stream. Sampling was conducted on two sampling dates (November and July) and the study included both amplicon and metagenomic sequencing in order to assess both the taxonomic and functional gene composition of the particle-associated bacterial assemblages. Effluent input resulted in increased FPM concentration and increased nutrient content of FPM, which were both correlated with an increase in microbial metabolic activity (MMA) immediately downstream of the WWTP. Effluent was a significant source of bacterial taxa to the stream, and there was a significant decrease in the diversity of the bacterial assemblages associated with the fine particles immediately downstream, as well as changes in their taxonomic and functional gene profiles. Taxa associated with wastewater treatment (Rhodocyclaceae and Xanthomonadaceae) and the metabolism of anthropogenic contaminants (Sphingobacteriales), and genes associated with multidrug efflux pumps and denitrification were more abundant immediately downstream. The effect of effluent on the taxonomic composition of the bacterial assemblages was much stronger than the effect on functional gene profiles, highlighting the functional redundancy within these communities. In addition, all the effects of effluent were short-lived and decreased with distance downstream, especially in November when the upstream flow moderated the effects of effluent. This study indicates that WWTP effluent impacts natural microbial communities by altering environmental conditions and sourcing new microbes but also demonstrates the functional redundancy and resilience of these communities.

## 1 Introduction

Wastewater Treatment Plants (WWTPs) are critical for the protection of freshwater ecosystems because they can reduce the negative impacts on aquatic ecosystems and human health that would arise from the direct release of large volumes of untreated wastewater. WWTPs can be effective for reducing biochemical oxygen demand (BOD), inorganic nutrient concentrations, and pathogen load, but WWTPs still represent important point-sources of nutrients, organic matter, and microbes to the environment (Martí et al., 2004; Mussmann et al., 2013). WWTPs frequently discharge effluent water to lotic ecosystems, and in many cases WWTP effluent makes up a significant proportion of the flow of the receiving water body (Brooks et al., 2006). For example, WWTP effluent accounts for greater than 50% of the flow of more than 900 rivers in the United States (Rice and Westerhoff, 2017). The impact of WWTP effluents can be especially significant in semiarid regions where streams have low dilution capacity because they can flow intermittently (Martí et al., 2004; Arnon et al., 2015). Because climate change is expected to decrease river flows and increase the number of intermittent streams in many parts of the world (Döll and Schmied, 2012), the number of WWTP effluent-dominated rivers is likely to increase worldwide.

Previous studies have documented the potential ecosystem effects of WWTP effluent inputs, including increased organic matter (Ruggiero et al., 2006) and nutrient loading (Waiser et al., 2011) and eutrophication (Gücker et al., 2006). WWTP effluent can also contain a variety of contaminants, including pharmaceuticals and personal care products, disinfectants and detergents, pesticides, and industrial chemicals (Kuzmanović et al., 2015; Cizmas et al., 2015). Effluent inputs can thus alter the physical and chemical characteristics of receiving water bodies and serve as point sources of contaminants (Lorentz et al., 2025), which can have significant effects on organisms living in these ecosystems (Carey and Migliaccio, 2009).

Microbes are important components of stream ecosystems due to the critical roles they play in various ecosystem processes, including organic matter decomposition and nutrient cycling (Pusch et al., 1998; Hall and Tank, 2003). Bacterial assemblages in streambed sediments are especially significant in streams because their abundance is generally much higher than that in the overlying water column (Sander and Kalff, 1993). Several previous studies have examined the effects of WWTP effluent on bacterial populations in streams (Garnier et al., 1992; Goñi-Urriza et al., 1999; Cébron et al., 2004) and have demonstrated that effluent can alter microbial community respiration (Ruggiero et al., 2006) and rates of nutrient cycling (Merseburger et al., 2011; Merbt et al., 2015; Bernal et al., 2018, 2020). For example, Lofton et al. (2007) reported a significant increase in denitrification rates in sediment samples collected downstream of a WWTP in Greensboro, NC, while Burdon et al. (2020) reported that microbial respiration and decomposition rates were positively influenced by WWTP inputs in an analysis of 20 streams in Switzerland. Effluent can also alter the taxonomic composition of stream microbial assemblages either through the direct input of organisms (Mussmann et al., 2013; Mansfeldt et al., 2020) or by altering stream physical or chemical characteristics to a degree that selects for different taxa (Drury et al., 2013). Lorentz et al. (2025) reported decreased abundance of photosynthetic organisms and shifts in benthic bacterial community composition in an effluent-receiving stream in the Chicago region; Wakelin et al. (2008) reported that WWTP effluent altered the composition of sediment bacterial communities in a rural stream in Australia; Merbt et al. (2015) reported shifts in the composition of epilithic ammonia oxidizing communities in La Tordera River (NE Spain) downstream of a WWTP; and Pascual-Benito et al. (2020) reported shifts in bacterioplankton communities immediately downstream of a WWTP in an intermittent stream in Catalonia, Spain.

Within the stream benthos, fine particulate matter (FPM) supports microbial activity because its high surface area to volume ratio promotes microbial colonization, decomposition of organic matter, nutrient cycling, and formation of new microbial biomass (Mendoza-Lera et al., 2017; Tank et al., 2010; Newbold et al., 2005). WWTPs can be point sources of nutrient-rich fine particles to streams, which can stimulate microbial activity in the receiving streams. For example, Drummond et al. (2022) reported an increase in nutrient rich fine particles downstream of a WWTP in La Tordera River (NE Spain) which were able to sustain high rates of microbial metabolic activity (MMA). However, the effect of WWTP effluents on the composition of microbial communities specifically associated with benthic FPM in rivers has not been studied. The present work sought to address this knowledge gap.

Our study analyzed FPM quantity, nutrient content, associated microbial activity, and bacterial assemblage composition in samples collected from sites upstream and downstream of the WWTP effluent input into La Tordera River, an intermittent Mediterranean stream in Spain. The study included both amplicon and metagenomic sequencing in order to assess both the taxonomic and functional gene composition of the bacterial assemblages. The objective was to assess the impact of the WWTP effluent input on bacterial assemblages associated with easily resuspended fine particles deposited in the streambed along an 800 m reach during two baseflow sampling times with contrasting hydrologic conditions. Specifically, the stream was dry upstream of the WWTP in July, while in November the stream was still at baseflow but was flowing upstream of the WWTP. The upstream flow in November provided dilution of the WWTP effluent and was a continuous source of FPM and microbes. We hypothesized that bacterial assemblages would differ in taxonomic and functional composition between upstream and downstream sites due to the influence of the WWTP effluent, and that this impact would be stronger and more persistent along the stream in July due to the lack of dilution from upstream flow and the lack of input of FPM associated bacteria from upstream sources. Testing these hypotheses will enhance our understanding of the influence of WWTP effluent on streambed microbial communities and their functional roles in receiving streams and on the persistence of the microbial footprint from the WWTP inputs along the stream.

## 2 Materials and methods

### 2.1 Field site

This study was conducted in a third-order stream reach of La Tordera River (NE of Barcelona, Spain) located immediately downstream of effluent input of the Santa Maria de Palautordera's WWTP (lat. 41.41′3.47″N, long. 2.27′33.19″W). At this location, the stream drains an 80 km^2^ watershed and the mean annual discharge is 267 ± 115 L s^−1^ (Merseburger et al., 2005). Effluent from the WWTP has an average discharge of 27 L s^−1^ that remains relatively constant throughout the year (Merseburger et al., 2005). The hydrologic regime of La Tordera River upstream of the WWTP is typical of an intermittent Mediterranean stream and it completely dries for several weeks during summer (Bernal et al., 2020). Samples were collected from a site 800 m upstream of the WWTP and from sites located 100, 300, 500, 600, 700, and 800 m downstream of the WWTP effluent input. The upstream and downstream reaches had similar streambed substrate composition characterized by rocks (10%), cobbles (60%), gravel (15%), and fine sediments (15%), and were flanked by a dense canopy of riparian trees with some areas of sparse vegetation (ca. 15% of each reach). Samples were collected on two sampling dates, July 31 and November 9, 2017. In November the river was flowing upstream of the WWTP, so this sampling included the collection of samples at the upstream site in addition to the downstream sites. In July the river was not flowing upstream of the treatment plant, so the July sampling included only the downstream sites.

### 2.2 Sample collection and physicochemical characterization

At each sampling location on each sampling date, we collected three replicate samples of fine particles from the streambed sediment following a modified version of Petticrew et al. (2007) as described in Drummond et al. (2022). A 35 cm diameter bottomless bucket was pushed into the stream bed to form a seal and block the flow of the surrounding water. The sampling water column depth in the bucket was recorded, and the top 3 cm of sediment was agitated by hand to re-suspend the fine particles into the water within the bucket. After a 10 s settling period a water sample was collected from the bucket using a 1 liter wide-mouth Nalgene bottle. The settling period allowed for the majority of the sand-sized sediment to settle out of the water, such that only material less than approximately 100 μm was sampled. This process was repeated for each of the three replicate samples from each site. This sampling approach included both FPM-associated and planktonic bacteria, so it is possible that variations in the depth of water at each of the sites may have impacted the relative contribution of planktonic bacteria from the water column to the subsequent analyses. However, bacteria are generally several orders of magnitude more abundant in sediments than in the overlying water column (Sander and Kalff, 1993), so the contribution of planktonic bacteria to our analysis was likely minor.

At each sampling location on each sampling date, we also collected stream water (from the thalweg) with pre-acid-washed polyethylene bottles after triple rinsing them with stream water. All fine particle and water samples were immediately placed on ice and covered from sunlight for transport to the lab. At each sampling location on each sampling date, we also measured on site stream water dissolved oxygen concentration (DO, in mg L^−1^ and % saturation) and water temperature (*T*, in °C) with a YSI ProODO device, and wetted width (*w*, in m). The average w for all sampling locations was used to provide a mean value for the reach. Stream discharge (*Q*, in L s^−1^) was measured with the cross-sectional method at the top of each reach that consists of measurements of velocity and depth across a representative cross sectional transect (Gordon et al., 2004).

### 2.3 Sample analysis

We quantified fine particulate matter (FPM) and fine particulate organic matter (FPOM) in each fine particle sample using the ash-free dry mass method (APHA, 2012). A known volume of each sample (~100–200 ml) was filtered onto a pre-ashed and pre-weighed 0.7 μm pore-size glass fiber filter (GF/F, Whatman, UK). Filters were dried at 50 °C for at least 24 h to reach a stable dry weight and that weight minus the weight of the filter was recorded as FPM. The filters were then placed in the muffle furnace at 500 °C for 5 h and then at 50 °C for 24 h. The mass loss was recorded as FPOM. The %OM of the particles was calculated as FPOM/FPM × 100. To standardize the FPM and FPOM measurements at the different field locations, the concentration of particles in the buckets (g ml^−1^) was converted to mass per stream surface area (g cm^−2^) by multiplying by the total water volume within the sampling bucket (ml) and then dividing by the streambed surface area sampled with the bucket. Total carbon (C) and nitrogen (N) content of the fine particles were determined using a CN analyzer (Thermo Finnigan Flash EA 1112, Waltham, MA, U.S.A.) and expressed as percentage of carbon (%C) and nitrogen (%N) in the dry mass. The C:N molar ratio was also calculated in each case. Water samples were filtered through pre-ashed GF/F and stored at 4 °C until laboratory analysis. Concentrations of nitrate (${\text{NO}}_{3}^{-}$), ammonium (${\text{NH}}_{4}^{+}$), and nitrite (${\text{NO}}_{2}^{-}$) in water samples were determined following standard colorimetric methods (APHA, 2012) on an autoanalyzer (FUTURA, Frepillon, France) at the LabQA analytical service of the CEAB-CSIC.

### 2.4 Microbial metabolic activity

Microbial metabolic activity (MMA in 1 h^−1^) in each fine particle sample was quantified within 24 h of the sampling date using the resazurin (Raz)–resorufin (Rru) system (Haggerty et al., 2009), in which the reduction of Raz to Rru is used as an indicator of aerobic metabolic activity (Haggerty et al., 2008; González-Pinzón et al., 2012). Prior to the Raz addition, each sample was thoroughly mixed and 40 ml of the sample was added to a glass scintillation vial, which was kept in a cool, dark area until the start of the assay. An injectate of Raz (0.022 g l^−1^) was freshly prepared and an aliquot of 400 μl was added to each vial. The vials were covered to avoid light contamination and shaken every 5 min throughout the assay to ensure sufficient mixing of the contents. A total of four subsamples were collected from each vial during the 3-h incubation. At each sampling time, the vials were first thoroughly shaken to homogenize the particulates, and 4 ml was pulled up from each vial and filtered through a 0.7 μm GF/F filter. The first 1 ml was displaced, and the 3 ml remaining was placed in a new sample vial in the dark until the fluorescence was tested on the spectrofluorometer. Immediately prior to analyzing each sample on the spectrofluorometer, 0.3 μl of buffer solution (pH 8) was added to each sample (Haggerty et al., 2008). Fluorescence of Raz and Rru were measured on a Shimadzu (Kyoto, Japan) RF spectrofluorometer. The excitation and emission wavelengths for Raz were 602 nm and 616 nm, while Rru were 571 nm and 585 nm (Bueno et al., 2002). Samples were placed in a quartz cuvette and held within the sample chamber of the spectrofluorometer for less than 1 min to minimize temperature changes (Haggerty et al., 2008). MMA was calculated from the transformation of Raz to Rru over time. The normalized Resazurin turnover (as a ratio between Rru and Raz at the specific sampling times) was computed from measured Raz and Rru concentrations according to (Haggerty et al., 2009).

### 2.5 Bacterial assemblage analysis

DNA was extracted from FPM samples using the PowerSoil DNA Isolation Kit (MO BIO, Carlsbad, CA, USA) according to the manufacturer's instructions. DNA samples were shipped at −20 °C to Loyola University Chicago for amplicon sequencing and to the Department of Public Health Microbiology of the National Laboratory of Health, Environment and Food (Ljubljana, Slovenia) for shotgun metagenomic sequencing. For amplicon sequencing, partial 16S rRNA genes were amplified using primers 515F and 806R, which amplify the V4 hypervariable region (Caporaso et al., 2011), and amplification was confirmed by agarose gel electrophoresis. The V4 region was chosen because it provides species richness estimates similar to those obtained with nearly full-length 16S rRNA genes (Youssef et al., 2009). Equimolar amounts of amplicons from each sample were sequenced in a 2 × 250 bp paired-end format using an Illumina MiSeq (Caporaso et al., 2012) by the DNA Services Facility, University of Illinois at Chicago. Raw amplicon sequence data from this study have been posted to the National Center for Biotechnology Information (NCBI) Sequence Read Archive (SRA) with accession number PRJNA1451225. For shotgun metagenomic sequencing, DNA libraries were prepared using the Illumina DNA Prep kit (formerly Nextera DNA Flex; Illumina, San Diego, CA, USA) according to the manufacturer's protocol. Briefly, genomic DNA was simultaneously fragmented and tagged with adapter sequences using bead-linked transposomes (tagmentation), followed by limited-cycle PCR amplification to incorporate dual indices and complete adapter sequences. Library quality and fragment size distribution were assessed using an Agilent 2100 Bioanalyzer and DNA concentration was quantified using Qubit dsDNA HS Assay (ThermoFisher Scientific). Libraries were normalized, pooled in equimolar concentrations, and sequenced on an Illumina MiSeq platform using a paired-end 2 × 250 bp configuration. Metagenomic sequence data are available at the European Nucleotide Archive (ENA) with accession number (PRJEB111230).

The 16S amplicon sequences were processed using mothur v.1.45.3 (Schloss et al., 2009) following the MiSeq Standard Operating Procedure (Kozich et al., 2013). Briefly, paired reads were assembled and demultiplexed, and any sequences with ambiguities or homopolymers > 8 bases were removed. Sequences were aligned using the SILVA-compatible alignment database available within mothur. Chimeric sequences were removed using VSEARCH (Rognes et al., 2016). Sequences were classified to the lowest possible taxonomic rank by comparison to the RDP training set using a naive Bayesian classifier (Wang et al., 2007) and a cutoff bootstrap value of 80%. Any unknown (i.e., not identified as bacterial), chloroplast, mitochondrial, archaeal, and eukaryotic sequences were removed. To avoid biases associated with uneven numbers of sequences across samples, the entire dataset was randomly subsampled (i.e., rarefied) to 27,611 sequences per sample (Schloss, 2023). Sequences were clustered into operational taxonomic units (OTUs) based on 97% sequence identity. Good's coverage was used to estimate the percentage of each of the assemblages that was covered by the subsampled sequence data set (Good, 1953), and this metric indicated that coverage averaged 94% across all samples (range 91%−97%). Diversity for each sample was quantified based on taxonomic richness (i.e., total number of OTUs observed) and the Shannon index (Shannon, 2001) calculated using mothur. To identify effluent-derived taxa, the uniquegroups parameter within the get.sharedseqs command in mothur was used to identify OTUs that were found in the 100 m downstream samples (July and November data combined) but were absent from the upstream samples.

Metagenomic sequence data were analyzed using the MG-RAST pipeline v3.0 (Meyer et al., 2008). Raw reads were subjected to automated quality control within MG-RAST, including removal of artificial replicate sequences, quality filtering to eliminate low-quality reads (based on read length and ambiguous base calls), and dereplication. Putative protein-encoding genes (PEGs) were identified using a BLASTX similarity search against the SEED non-redundant database, applying MG-RAST default parameters (maximum e-value cutoff of 1 × 10^−5^, minimum alignment length of 15 amino acids, and minimum identity threshold of 90%). Functional annotation was assigned according to the hierarchical SEED subsystems classification, enabling analysis at multiple levels of functional organization.

Gene abundance profiles were generated based on the number of reads assigned to each functional category. To account for differences in sequencing depth across samples, counts were normalized to relative abundances prior to downstream analysis. Comparative analyses of functional profiles were performed across samples using normalized abundance tables exported from MG-RAST. Multivariate analyses, including ordination methods, were used to explore similarities and differences in functional composition between samples.

### 2.6 Statistical analyses

We assessed differences in measured variables (FPM, FPOM, MMA, species richness, Shannon index, relative abundance of bacterial families, and relative abundance of functional genes) based on two factors, sampling location and date, using Systat v13. In all cases, differences were considered statistically significant if *p* < 0.05. To assess how persistent the influence of the WWTP effluent input on these variables was along the stream, data from downstream sites for both sampling dates (July and November) were analyzed by two-way ANOVA with sampling location and date as factors. In addition, data for all sites from the November sampling date, including the upstream site, were compared by one-way ANOVA with location as the factor. This second test allowed us to assess the impact of WWTP effluent input on the chemical and microbial footprint sourced from upstream. For both two-way and one-way ANOVAs, pairwise differences between locations were assessed based on Tukey's HSD Test. Prior to running ANOVAs normality of data was assessed according to the Shapiro–Wilk test and data that were not normally distributed were transformed. FPM and FPOM concentrations and MMA rates were not normally distributed (*p* < 0.001 for all three variables), so these data were log transformed prior to running ANOVAs. Richness and Shannon index data were normally distributed (*p* = 0.387 and 0.255, respectively) so these data were used directly for ANOVAs. Bacterial family abundance data that were normally distributed were used directly for ANOVAs, while family abundance data that were not normally distributed were log transformed prior to running ANOVAs. Because the OM, C, and N content of the FPM are reported as percentages, these data were arcsine square root transformed prior to running ANOVAs. Correlations between MMA and FPM characteristics (FPM abundance, FPOM abundance, %OM, %C, and %N) were assessed by determining Pearson correlation coefficients and Bonferroni-corrected probabilities using Systat v13. Data from all sites and sampling dates were included in the analyses of correlations.

The taxonomic composition of bacterial assemblages from each sample location on the two sampling dates was compared by calculating dissimilarities for each pair of samples based on the Bray Cutis index (Bray and Curtis, 1957). Statistical significance of differences in assemblages between sampling locations and sampling dates based on the Bray Cutis index was assessed by analysis of molecular variance (AMOVA) (Excoffier et al., 1992) run in mothur. The Bray Cutis dissimilarity matrix was visualized using principal coordinates analysis (PCOA) run in R Statistical Software (v4.3.1; R Core Team, 2020). The correlations between bacterial assemblage composition and environmental factors were analyzed using the envfit function in R. The environmental factors were overlaid on the PCOA ordination as vectors, with the direction and length of each vector indicating the direction and intensity of the correlation with the PCOA axes. Metastats analysis (White et al., 2009) run in mothur was used to identify OTUs with the largest differences in relative abundance between the sites close to and the sites far from the effluent input and to identify OTUs with the largest differences in relative abundance between the November and July sampling dates.

Gene abundance values produced from the metagenome data were normalized to the number of reads per sample. Relative abundances of specific genes or gene families were compared across sites by one-way ANOVA run using Systat v13 with location as the factor. Prior to running ANOVAs normality of data was assessed according to the Shapiro–Wilk test and data that were not normally distributed were log transformed. When ANOVA revealed a significant location effect (*p* < 0.05) pairwise differences between locations were assessed based on Tukey's HSD Test. Dissimilarities in full functional gene profiles for each pair of samples were determined based on the Bray Cutis index (Bray and Curtis, 1957). The statistical significance of differences in functional gene profiles between sampling locations and sampling dates based on the Bray Cutis index was assessed by Adonis (McArdle and Anderson, 2001). The Bray Cutis dissimilarity matrix was visualized using principal coordinates analysis (PCOA). The correlations between functional gene profiles and environmental factors were analyzed using the envfit function in R. The environmental factors were overlaid on the PCOA ordination as vectors, with the direction and length of each vector indicating the direction and intensity of the correlation with the PCOA axes. Correlations between Bray Curtis dissimilarity scores for taxonomic and functional gene profiles for all sites and sampling dates were assessed by determining Pearson correlation coefficients and Bonferroni-corrected probabilities using Systat v13.

## 3 Results

### 3.1 FPM characterization: quantity, nutrient content, and associated microbial metabolic activity

**Figure 1:**
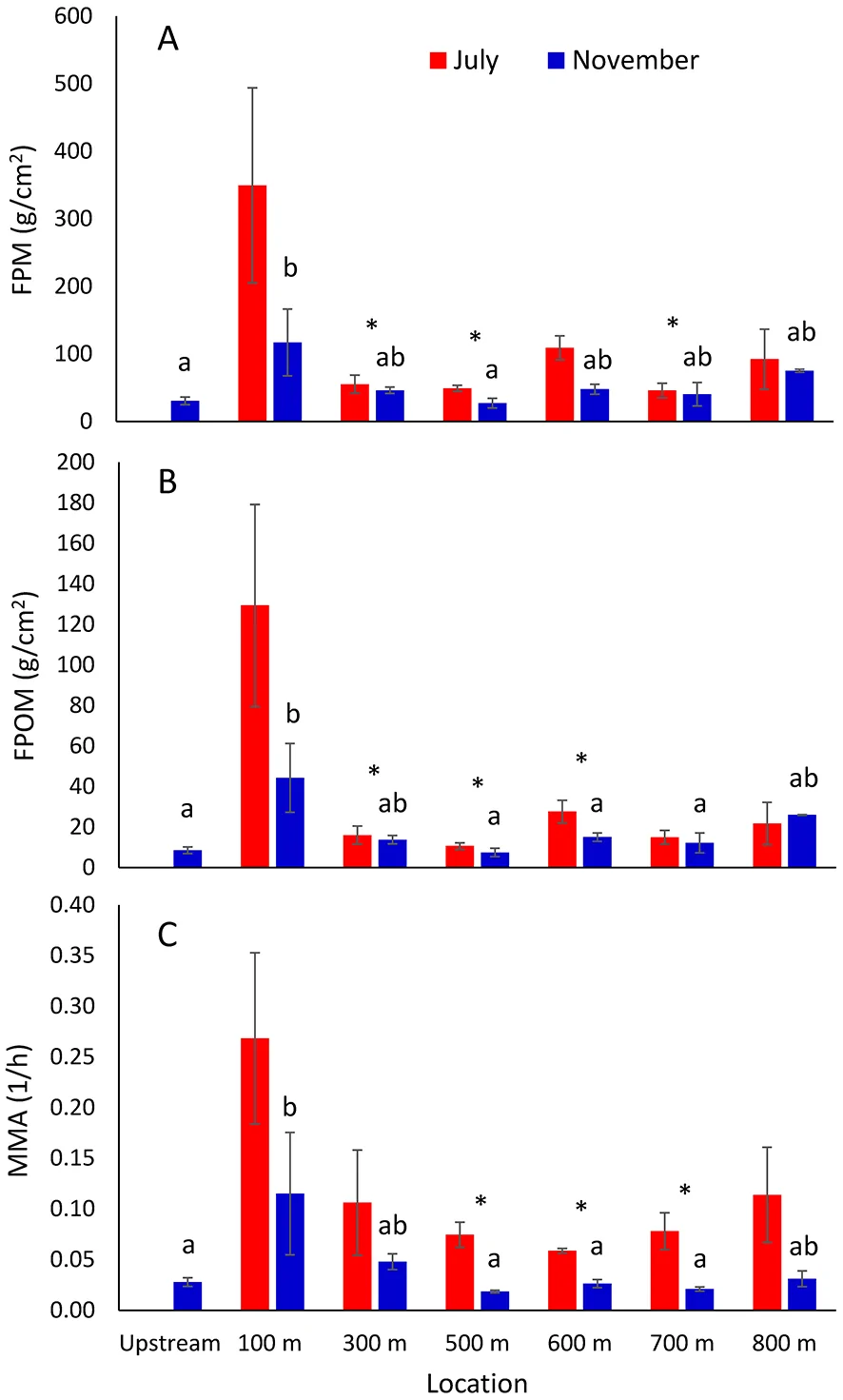
Spatial patterns of **(A)** fine particulate matter (FPM), **(B)** fine particulate organic matter (FPOM) and **(C)** microbial metabolic activity (MMA) upstream and at several distances downstream of the wastewater treatment plant effluent input. Data represent mean values (*n* = 3 for downstream sites and *n* = 6 for upstream) ± standard error. Two-way ANOVA of downstream samples indicated a significant effect of sampling location on FPM (*p* = 0.0046), but no effect of sampling date (*p* = 0.0503) and no interaction (*p* = 0.514); the same pattern was observed for FPOM (*p* < 0.001, *p* = 0.148, and *p* = 0.380 for sampling location, sampling date, and interaction, respectively); for MMA, a significant effect was found for sampling location (*p* = 0.002) and sampling date (*p* < 0.001) but not for the interaction (*p* = 0.86). For these tests, asterisks indicate locations with values significantly lower than the 100 m site. One-way ANOVA for November samples indicated a significant effect of sampling location on FPM (*p* = 0.018), FPOM (*p* = 0.002), and MMA (*p* = 0.003). For these tests, different lower-case letters indicate significant differences between locations.

The input of effluent from the WWTP resulted in increased concentrations of FPM and FPOM in the streambed sediment of La Tordera River (Figures 1A, B). The concentration of FPM and FPOM peaked at the site closest to the effluent input (100 m), especially in July when the river was not flowing upstream of the WWTP. However, in November, both FPM and FPOM were significantly higher 100 m downstream of the WWTP effluent compared to the upstream site, indicating that the WWTP was a point source of FPM to the river. During both July and November, FPM and FPOM concentrations were generally lower further downstream than at 100 m.

Effluent input also resulted in an increase in the nutrient content of the FPM in the streambed (Table 1). Specifically, sampling location had a significant effect on %OM (*p* < 0.001), %C (*p* < 0.001), and %N (*p* < 0.001) of the FPM. These three variables were all significantly higher (*p* < 0.001) at the site closest to the effluent input (100 m) than (i) at all of the other downstream sites during both July and November, and (ii) at the upstream site in November (*p* < 0.001). Sampling date also had a significant effect on %OM (*p* < 0.001), %C (*p* < 0.001), and %N (*p* = 0.002) of the FPM, with percent organic matter slightly higher and percent carbon and percent nitrogen both slightly lower in November (Table 1), when the river was flowing upstream of the WWTP. There were also large differences in stream water chemistry between the upstream and downstream sites in November, with concentrations of oxygen and nitrate being lower downstream and nitrite and ammonium concentration being higher downstream (Table 2). The concentrations of oxygen, nitrate and ammonium remained generally consistent along the downstream reach sites for the November sampling. There were no consistent differences in these water chemistry parameters between the July and November sampling dates.

**Table 1:** Chemical characterization of fine particulate matter in streambeds upstream and at several distances downstream of the wastewater treatment plant effluent input.

Site	%OM		%C		%N	
	July	November	July	November	July	November
Upstream	NA	28.2 ± 0.7	NA	0.70 ± 0.02	NA	8.0 ± 0.1
100 m	37.7 ± 1.2	39.2 ± 1.7	1.66 ± 0.08	1.19 ± 0.16	15.5 ± 0.6	11.5 ± 1.6
300 m	28.7 ± 1.4	29.6 ± 1.8	0.89 ± 0.06	0.66 ± 0.04	8.5 ± 0.8	7.0 ± 0.3
500 m	21.5 ± 1.3	27.4 ± 0.4	0.71 ± 0.05	0.58 ± 0.00	7.2 ± 0.7	5.9 ± 0.5
600 m	25.1 ± 0.9	31.8 ± 0.5	0.78 ± 0.05	0.66 ± 0.01	8.2 ± 0.6	6.9 ± 0.3
700 m	32.6 ± 0.7	30.9 ± 2.0	0.96 ± 0.07	0.66 ± 0.05	9.8 ± 1.1	6.7 ± 0.7
800 m	24.3 ± 1.0	34.7 ± 1.1	0.82 ± 0.12	0.78 ± 0.07	8.4 ± 1.8	7.8 ± 0.8

Data represent mean values (*n* = 3 for downstream sites and *n* = 6 for upstream) ± standard error.

%OM, percent organic matter; %C, percent carbon; %N, percent nitrogen.

**Table 2:** Stream water chemistry upstream and at several distances downstream of the wastewater treatment plant effluent input.

Site	Temperature (°C)		O_2_ (mg L^−1^)		O_2_ (%)		NH_4_-N (mg L^−1^)		NO_3_-N (mg L^−1^)		NO_2_-N (mg L^−1^)		DIN-N (mg L^−1^)	
	July	November	July	November	July	November	July	November	July	November	July	November	July	November
Upstream	NA	18.2	NA	8.73	NA	92.9	NA	0.015	NA	2.328	NA	0.002	NA	2.346
100 m	27.0	22.6	4.09	4.77	51.1	55.0	2.675	1.612	0.917	1.227	0.188	0.082	3.779	2.921
300 m	25.9	21.9	3.86	4.38	47.5	49.8	2.675	1.988	1.496	1.014	0.250	0.119	4.420	3.121
500 m	25.7	21.4	4.08	4.71	49.9	53.1	1.702	1.949	1.798	1.773	0.253	0.250	3.753	3.971
600 m	26.0	21.2	5.09	4.75	62.5	53.3	1.442	2.103	2.514	1.868	0.315	0.315	4.271	4.286
700 m	25.2	20.9	4.80	4.54	56.7	50.5	0.524	1.896	2.528	2.698	0.259	0.382	3.310	4.977
800 m	24.3	20.3	6.62	4.60	78.1	50.9	0.195	2.120	2.070	1.939	0.102	0.318	2.366	4.378

Data represent individual readings for each site and sampling time.

Effluent input increased microbial metabolic activity (MMA) associated with FPM in the stream (Figure 1C). MMA varied significantly with sampling location, being highest at the site closest to the effluent input (100 m) and decreasing with distance downstream. In November, MMA was higher 100 m downstream as compared to upstream, indicating that effluent input stimulated MMA associated with FPM in the stream. MMA also varied significantly with sampling date, being consistently higher in July, when the river was not flowing upstream of the WWTP, than in November, when the river was flowing upstream. Stream water temperature was higher in July than November (Table 2), which may have also contributed to higher MMA.

Several characteristics of FPM were significantly correlated with the associated MMA. MMA was positively correlated with the concentration of both FPM (*r* = 0.493, *p* = 0.001; Supplementary Figure S1A) and FPOM (*r* = 0.497, *p* = 0.001; Supplementary Figure S1B), and with the nutrient content of the FPM, showing positive correlations with %C (*r* = 0.758, *p* < 0.001; Supplementary Figure S2A) and %N (*r* = 0.769, *p* < 0.001; Supplementary Figure S2B), but no correlation with %OM (*r* = 0.293, *p* = 0.063; Supplementary Figure S2C). These data indicate that the increases in organic matter and nutrient content of the FPM downstream of the effluent input were linked to an increase in MMA, but that these effects decreased with distance from the input. Moreover, these effects were generally stronger in July, when the stream was not flowing upstream of the WTTP, than in November when the upstream flow could dilute the effluent.

### 3.2 Taxonomic analysis of bacterial assemblages associated with FPM in the streambed

**Figure 2:**
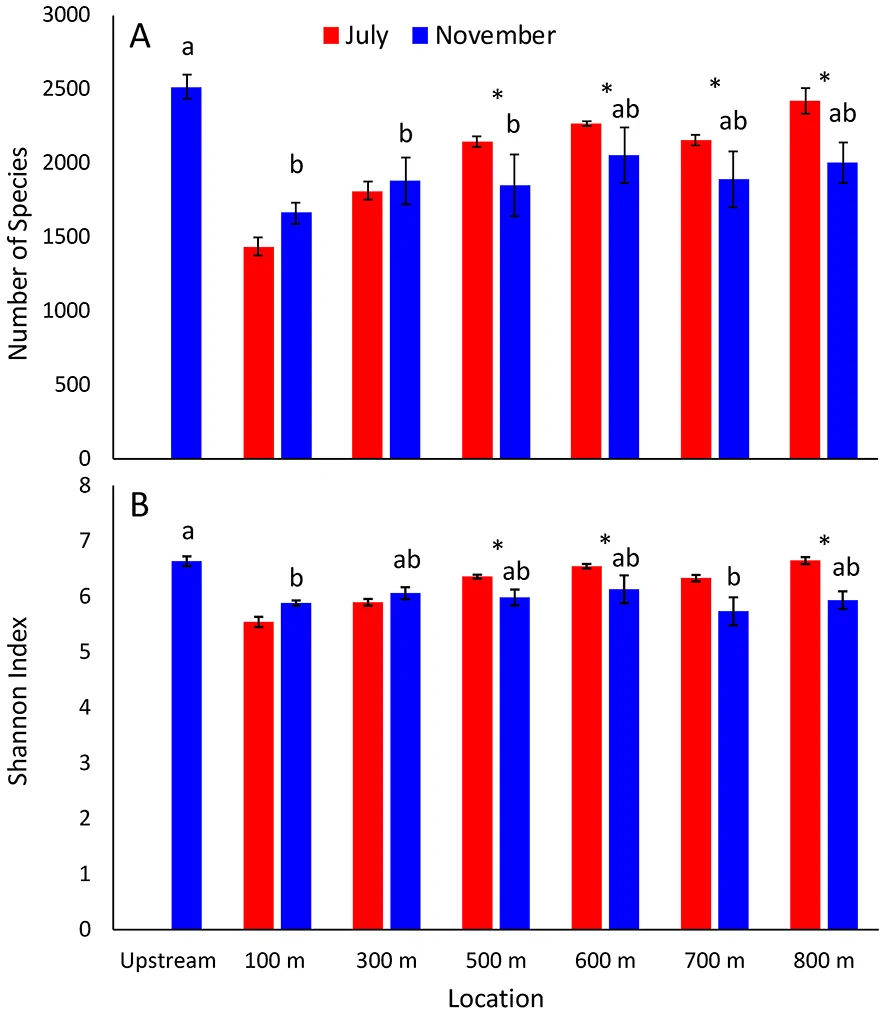
Spatial patterns of **(A)** taxonomic richness and **(B)** Shannon diversity of bacterial assemblages associated with fine particular matter upstream and at several distances downstream of the wastewater treatment plant effluent input. Data represent mean values (*n* = 3 for downstream sites and *n* = 6 for upstream) ± standard error. Two-way ANOVA of downstream samples indicated a significant effect of location on richness (*p* < 0.001) but no effect of date (*p* = 0.061) and no interaction (*p* = 0.126). One-way ANOVA for November samples indicated a significant effect of location on richness (*p* = 0.005). Two-way ANOVA of downstream samples indicated significant effects of location (*p* < 0.001) and date (*p* = 0.002) on diversity and a significant interaction (*p* = 0.002). One-way ANOVA for November samples indicated a significant effect of location on diversity (*p* = 0.004). Asterisks indicate locations with values significantly higher than the 100 m site. Different lower-case letters indicate significant differences between locations for November samples.

Taxonomic richness (Figure 2A) and Shannon diversity (Figure 2B) of bacterial assemblages associated with FPM varied with sampling location. Richness and diversity were lower at the sites closest to the WWTP effluent input (100 m and 300 m) compared to sites located further downstream (from 500 to 800 m from the effluent input). This spatial pattern was similar in July and November. In November, richness and diversity were highest at the upstream site, were significantly lower at 100 m downstream of the effluent input, and then gradually increased with downstream distance, reaching a level equivalent to the upstream site at 600 m for richness and at 300 m for diversity. These results indicate a negative effect of WWTP effluent on the taxonomic richness and diversity of bacterial assemblages associated with FPM, and a recovery of richness and diversity with distance from the effluent input. Richness did not vary significantly with sampling date, but diversity did vary significantly with sampling date, with July samples having higher diversity on average than November samples.

**Figure 3:**
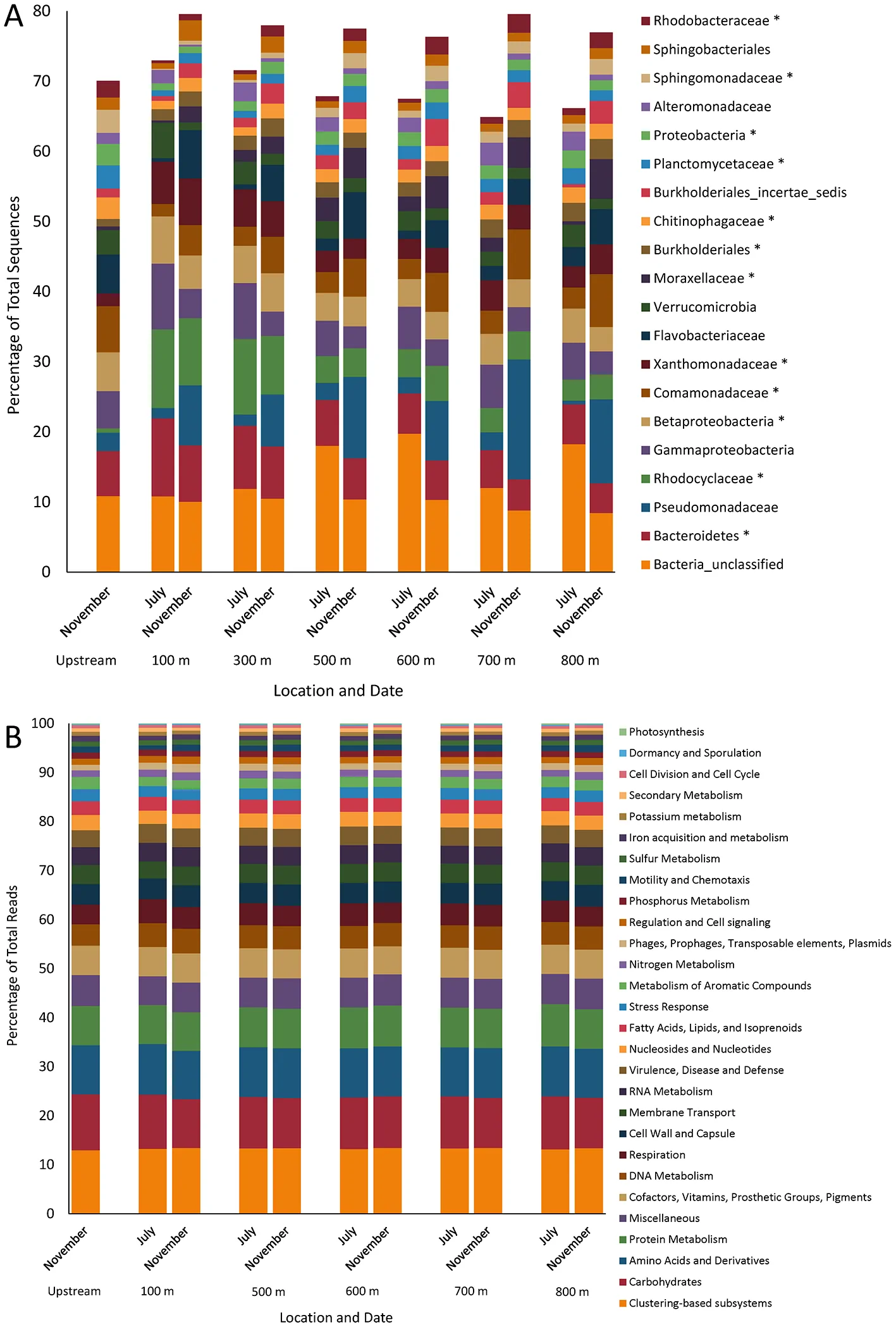
Spatial patterns of average relative abundance of **(A)** the 20 most abundant bacterial families and **(B)** functional genes at the subsystem level within bacterial assemblages associated with fine particular matter upstream and at several distances downstream of the wastewater treatment plant effluent input. Data represent mean values (*n* = 3 for downstream sites and *n* = 6 for upstream). For panel **(A)**, asterisks (*) indicate significant differences based on sampling location (ANOVA *p* < 0.05).

In order to provide a broad overview of the composition of the bacterial assemblages associated with FPM at the sites, we focused on the 20 most abundant bacterial families that together comprise between 65 and 80% of total sequences (Figure 3A). These most abundant families include an unclassified Bacterial family, a Bacteroidetes family, Pseudomonadaceae, Rhodocyclaceae, a Gammaproteobacteria family, a Betaproteobacteria family, Comamonadaceae, and Flavobacteriaceae. The abundance of 12 of these families varied significantly with sampling location (Figure 3A). Three of these 12 families were significantly more abundant at the 100 m downstream site than at the upstream site, including Bacteroidetes, Rhodocyclaceae, Xanthomonadaceae, indicating a positive effect of effluent on these taxa. For the November sampling, these three bacterial families accounted for 6.4%, 0.6% and 1.9% of total sequences at the upstream site compared to 8.1%, 9.7%, and 6.6% of total sequences at the 100 m site. In addition, the relative abundances of these three families decreased with distance from the effluent input, indicating that the positive effect of the effluent on the abundance of these taxa decreased with distance. In contrast, six of the families whose abundances varied significantly with sampling location were significantly less abundant at the 100 m downstream site than at the upstream site, including Comamonadaceae, Chitinophagaceae, Planctomycetaceae, Proteobacteria, Sphingomonadaceae, and Rhodobacteraceae, indicating a negative effect of effluent on these taxa. The largest decreases were observed for Comamonadaceae, which decreased from 6.6% upstream to 4.4% at 100 m, and Sphingomonadaceae, which decreased from 3.3% to 0.6%, but the relative abundances of both of these families increased with distance from the effluent input, reaching 7.6% and 2.2%, respectively, at 800 m.

**Figure 4:**
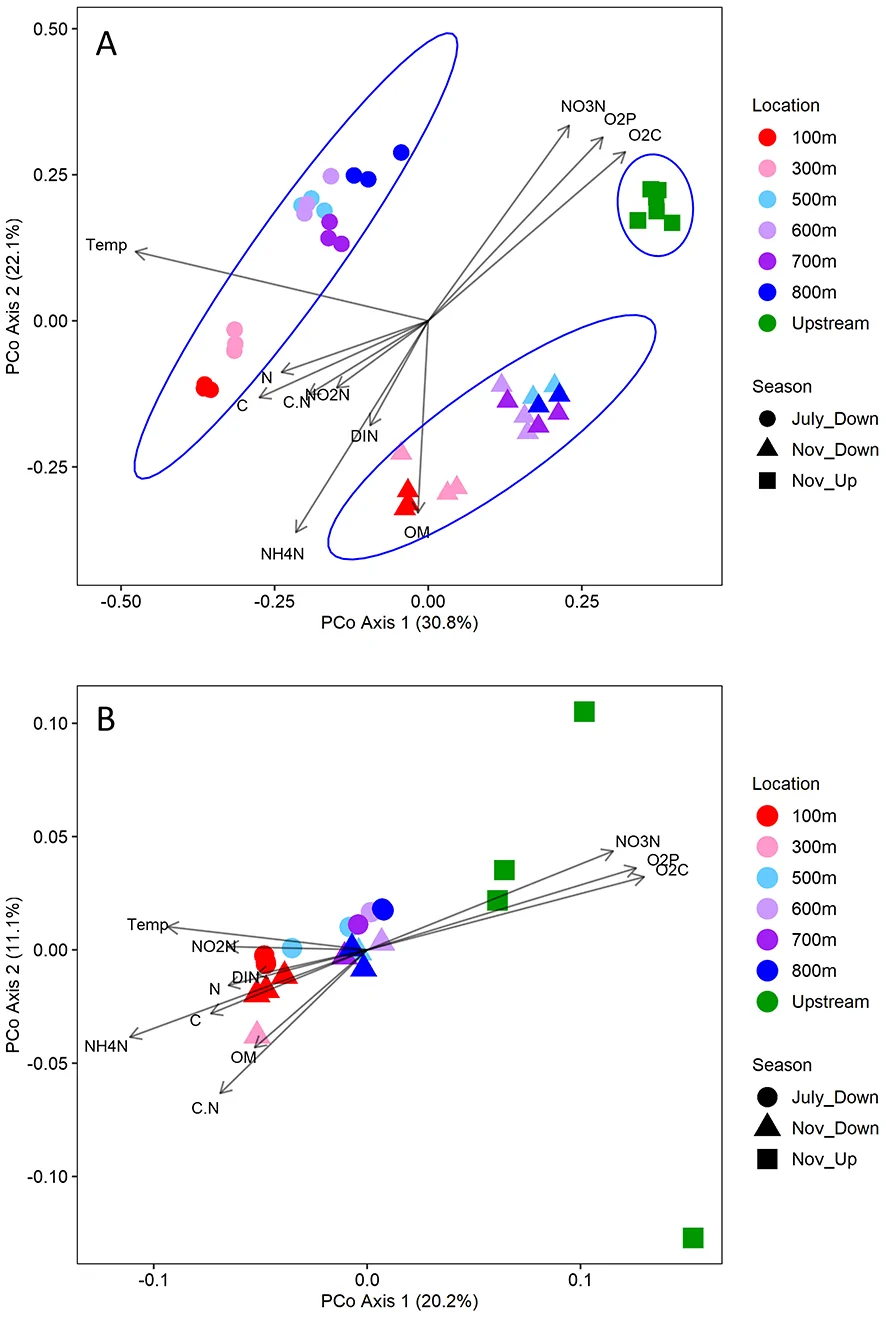
Beta diversity based on **(A)** taxonomy profiles and **(B)** functional gene profiles of bacterial assemblages associated with fine particular matter upstream and at several distances downstream of the wastewater treatment plant effluent input. Principal coordinates analysis (PCOA) ordinations were based on the Bray Cutis dissimilarity index. The variance explained by each PCOA axis is shown in parenthesis in the axis labels. Vectors represent correlations between environmental variables and PCOA axes. Environmental variables are abbreviated as follows: sediment organic matter (OM), FPOM percent carbon (C). FPOM percent nitrogen (N), FPM carbon to nitrogen ratio (C:N), water temperature (Temp), dissolved oxygen concentration (O_2_C), dissolved oxygen percentage (O_2_P), ammonium (NH_4_N), nitrate (NO_3_N), nitrite (NO_2_N), dissolved inorganic nitrogen (DIN). The ovals surrounding the groups in panel A represent the 95% confidence interval for the November Upstream (Nov_Up), November Downstream (Nov_Down), and July Downstream (July_Down) samples.

Analysis of the beta diversity of the FPM-associated bacterial assemblages at the OTU level via PCOA revealed three distinct clusters representing (1) upstream sites from November (green squares), (2) downstream sites from November (triangles), and (3) downstream sites from July (circles; Figure 4A). AMOVA confirmed that the differences in beta diversity between each of these three clusters were statistically significant (*p* < 0.001 for all pairwise comparisons of these three groups). There was also a separation of downstream sites that were close to the effluent input (100 m and 300 m; red and pink shapes) from those that were farther from the effluent input (500 m to 800 m; blue and purple shapes). AMOVA confirmed that the differences in taxonomic profiles between the near effluent (100 and 300 m) and far from effluent (from 500 to 800 m) groups were statistically significant, regardless of whether July and November samples were split or analyzed together (in all cases *p* < 0.001). In the ordination space, samples from the downstream sites close to the effluent input (red and pink shapes) were farther from the upstream samples (green shapes) than samples from the downstream sites more distant from the effluent input (blue and purple shapes). This pattern held during both July and November, indicating that the impact of effluent on bacterial taxonomy is strongest immediately downstream and decreases with distance downstream, with assemblages becoming more similar to those at the upstream site as you move further from the effluent input.

Incorporation of environmental variables into the PCOA ordination (Figure 4A) demonstrated that assemblages closer to the effluent input (100 m and 300 m; red and pink shapes) were positively correlated with concentrations of nutrients in both the FPM (FPOM, %C, %N, FPM C:N) and the water column, (ammonium and dissolved inorganic nitrogen), and negatively correlated with concentrations of oxygen and nitrate in the water column. These results suggest a relationship between the impact of WWTP effluent on the physicochemical conditions in the river (i.e., increased nutrient concentrations and decreased oxygen) and the taxonomic profiles of bacterial assemblages associated with FPM. Separation of July samples (circles) from November samples (triangles) was correlated with a difference in water temperature.

To determine if any bacterial OTUs were directly added to the river by the WWTP effluent, we identified bacterial OTUs that were detected at the 100 m downstream site but were not detected at the upstream site, i.e., “downstream-only OTUs”. There were 3,732 downstream-only OTUs which cumulatively accounted for 25% of the total sequences found at the 100 m downstream site, indicating that the effluent was a significant source of bacterial taxa to the river. Some of the most abundant downstream-only OTUs were assigned to the taxa Verrucomicrobia, Sphingobacteriales, Bacteroidetes, and Flavobacteriales (Supplementary Table S1).

Based on the distinct clustering of the near effluent and far from effluent assemblages revealed by the PCOA (Figure 4A), metastats analysis was used to identify specific OTUs that differed in relative abundance between these two groups (Table 3). Two Rhodocyclaceae OTUs, one Xanthomonadaceae OTU, and one Sphingobacteriales OTU were significantly more abundant in the near effluent samples. In contrast, two Pseudomonas OTUs, one Moraxellaceae OTU, and two Comamonadaceae OTUs were significantly more abundant in the far from effluent samples. One of these Comamonadaceae OTUs was also highly abundant in the upstream samples, representing more than 5% of all sequences. One Flavobacterium OTU was also highly abundant in the in the upstream samples, representing more than 3% of all sequences, but this taxon was not significantly different in relative abundance between the near effluent samples and the far from effluent samples.

**Table 3:** Relative abundances of bacterial OTUs with the largest differences in relative abundance between the sites close to and the sites far from the effluent input.

Bacterial OTUs	Near effluent^b, c^	Far from effluent^b, d^	*p*-value^e^	Upstream^f^
OTUs^a^ that were more abundant near effluent				
Rhodocyclaceae	3.86% ± 0.22%	1.92% ± 0.13%	0.000	0.23% ± 0.00%
Xanthomonadaceae	3.69% ± 0.44%	2.31% ± 0.11%	0.008	0.92% ± 0.01%
Rhodocyclaceae	2.55% ± 0.16%	1.18% ± 0.11%	0.000	0.00% ± 0.00%
Cellvibrio	1.35% ± 0.21%	0.42% ± 0.04%	0.001	0.00% ± 0.00%
Betaproteobacteria	1.33% ± 0.17%	0.44% ± 0.05%	0.000	0.04% ± 0.00%
Pseudomonadaceae	1.13% ± 0.11%	0.27% ± 0.03%	0.000	0.02% ± 0.00%
Aquabacterium	2.79% ± 0.34%	1.94% ± 0.20%	0.050	0.33% ± 0.00%
Flavobacterium	1.92% ± 0.42%	1.11% ± 0.13%	0.091	3.01% ± 0.13%
Sphingobacteriales	0.68% ± 0.11%	0.15% ± 0.02%	0.000	0.03% ± 0.00%
Lysobacter	1.05% ± 0.10%	0.53% ± 0.09%	0.001	0.06% ± 0.00%
Betaproteobacteria	0.56% ± 0.08%	0.06% ± 0.01%	0.000	0.01% ± 0.00%
OTUs^a^ that were more abundant far from effluent				
Flavobacterium	0.97% ± 0.25%	1.64% ± 0.27%	0.095	0.01% ± 0.00%
Rhodobacteraceae	0.55% ± 0.09%	1.27% ± 0.08%	0.000	1.40% ± 0.00%
Comamonadaceae	0.18% ± 0.06%	1.01% ± 0.12%	0.000	0.31% ± 0.00%
Aquabacterium	0.22% ± 0.10%	1.29% ± 0.25%	0.001	0.10% ± 0.00%
Comamonadaceae	2.23% ± 0.23%	3.72% ± 0.25%	0.001	5.38% ± 0.01%
Moraxellaceae	0.92% ± 0.18%	2.71% ± 0.40%	0.001	0.68% ± 0.01%
Pseudomonas	3.13% ± 0.42%	5.68% ± 0.84%	0.017	0.50% ± 0.00%
Pseudomonas	1.11% ± 0.20%	4.76% ± 1.00%	0.003	0.68% ± 0.01%

^a^OTUs were identified to the lowest possible taxonomic rank. In some cases multiple distinct OTUs were identified to the same family (e.g., Rhodocyclaceae, Comamonadaceae) or genus (e.g., Pseudomonas).

^b^Mean values ± standard error with July and November samples pooled together.

^c^Near effluent samples are 100 and 300 m from the effluent input.

^d^Far from effluent samples are 500, 600, 700 and 800 m from the effluent input.

^e^*p*-value for comparison of near effluent and far from effluent samples.

^f^Data for upstream were included for comparison.

Metastats analysis also indicated that several bacterial OTUs were significantly different in relative abundance between the November and July sampling dates (Table 4). Specifically, two Gammaproteobacteria OTUs, one Haliea OTU, and one Aeromonas OTU were significantly more abundant in the July samples. In contrast, two Pseudomonas OTUs, three Comamonadaceae OTUs, one Moraxellaceae OTU, and two Flavobacterium OTUs were significantly more abundant in the November samples. One of the Comamonadaceae OTUs and one of the Flavobacterium OTUs were also highly abundant in the November upstream samples, representing more than 5% and 3% of all sequences, respectively.

**Table 4:** Relative abundances of bacterial OTUs with the largest differences in relative abundance in downstream samples from the November and July sampling dates.

Bacterial OTUs	November^b^	July^b^	*p*-value^c^	Upstream^d^
OTUs^a^ that were more abundant in November				
Pseudomonas	4.78% ± 0.63%	0.02% ± 0.01%	0.001	0.50% ± 0.00%
Pseudomonas	3.47% ± 0.78%	0.00% ± 0.00%	0.001	0.68% ± 0.01%
Comamonadaceae	3.19% ± 0.25%	0.69% ± 0.07%	0.001	5.38% ± 0.01%
Moraxellaceae	2.08% ± 0.34%	0.01% ± 0.00%	0.001	0.17% ± 0.00%
Flavobacterium	1.40% ± 0.21%	0.12% ± 0.02%	0.001	0.01% ± 0.00%
Flavobacterium	1.40% ± 0.19%	0.24% ± 0.04%	0.001	3.01% ± 0.13%
Aquabacterium	2.24% ± 0.20%	1.15% ± 0.19%	0.001	0.33% ± 0.00%
Aquabacterium	0.91% ± 0.21%	0.11% ± 0.02%	0.001	0.10% ± 0.00%
Cellvibrio	0.75% ± 0.13%	0.01% ± 0.00%	0.001	0.55% ± 0.00%
Comamonadaceae	0.71% ± 0.13%	0.01% ± 0.00%	0.001	0.31% ± 0.00%
Rhodobacteraceae	1.02% ± 0.10%	0.36% ± 0.04%	0.001	1.40% ± 0.00%
Comamonadaceae	0.64% ± 0.10%	0.03% ± 0.01%	0.001	0.09% ± 0.00%
OTUs^a^ that were more abundant in July				
Comamonadaceae	1.05% ± 0.05%	1.60% ± 0.09%	0.001	1.22% ± 0.00%
Gammaproteobacteria	0.43% ± 0.03%	1.00% ± 0.09%	0.001	0.16% ± 0.00%
Moraxellaceae	0.40% ± 0.07%	1.03% ± 0.25%	0.012	0.14% ± 0.00%
Verrucomicrobia subdivision 3	0.08% ± 0.01%	0.78% ± 0.29%	0.002	0.00% ± 0.00%
Gordonia	0.05% ± 0.01%	1.09% ± 0.15%	0.001	0.00% ± 0.00%
Cyanobacteria	0.04% ± 0.01%	1.10% ± 0.28%	0.001	0.00% ± 0.00%
Bacteria unclassified	0.29% ± 0.05%	1.46% ± 0.26%	0.001	0.03% ± 0.00%
Aeromonas	0.26% ± 0.04%	1.71% ± 0.31%	0.001	0.08% ± 0.00%
Haliea	0.65% ± 0.07%	2.10% ± 0.12%	0.001	1.45% ± 0.00%
Gammaproteobacteria	0.39% ± 0.03%	2.61% ± 0.40%	0.001	0.12% ± 0.00%

^a^OTUs were identified to the lowest possible taxonomic rank. In some cases multiple distinct OTUs were identified to the same family (e.g., Comamonadaceae) or genus (e.g., Pseudomonas, Flavobacterium, Aquabacterium).

^b^Mean values ± standard error.

^c^*p*-value for comparison of downstream samples from the November and July sampling dates.

^d^Data for upstream were included for comparison.

### 3.3 Functional gene analysis of bacterial assemblages associated with FPM in the streambed

At the broad subsystem level (level 1, which represents major functional categories) there were no significant variations in the relative abundance of genes across sites or sampling dates (Figure 3B). Genes assigned to specific pathways relevant to WWTP impacts were also examined. Within the category of nitrogen metabolism (Supplementary Figure S3), the upstream site had a significantly lower abundance of genes involved in denitrification as compared to all of the downstream sites (*p* < 0.01). In addition, the 100 m site had a significantly higher abundance of genes involved in nitrogen fixation as compared to the upstream site (*p* < 0.05). Within the category of antibiotic resistance (Supplementary Figure S4), the 100 m site had a significantly higher abundance of genes encoding multidrug efflux pumps as compared to the upstream site (*p* < 0.05). There were no significant differences based on location for any of the other genes linked to antibiotic resistance that were identified in the samples (e.g., fluoroquinolone, methicillin, polymyxin and beta-lactam resistance genes). Finally, there were no significant differences based on location for any of the genes related to virulence that were identified in the samples (Supplementary Figure S5).

PCOA analysis of the bacterial functional gene profiles (Figure 4B) showed similar trends to those observed for the taxonomic profiles (Figure 4A). Specifically, analysis of functional gene profiles revealed distinct clustering of three groups of samples, i.e., (1) upstream samples from November (green squares), (2) downstream samples that were far from the effluent input (500 m to 800 m; blue and purple shapes), and (3) downstream samples close to the effluent input (100 m and 300 m; red and pink shapes). ADONIS confirmed that differences in functional gene profiles between each of these three clusters were statistically significant (*p* < 0.01 for all pairwise comparisons of these three groups). PCOA analysis of functional gene profiles also indicated a separation of downstream samples by sampling date, i.e., downstream samples from November (triangles) and downstream samples from July (circles; Figure 4B), and ADONIS confirmed that there was a significant difference in functional gene profiles between the July and November samples (*p* < 0.001). Incorporation of environmental variables into the functional gene PCOA ordination (Figure 4B) revealed the same trends that had been observed for the taxonomic profiles (Figure 4A). Namely, (i) the close to effluent samples (100 m and 300 m; red and pink shapes) were positively correlated with concentrations of nutrients in both the FPM and the water column, and negatively correlated with concentrations of water column oxygen and nitrate, and (ii) separation of July samples (circles) from November samples (triangles) was correlated with higher water temperature in July.

### 3.4 Comparison of taxonomic and functional gene profiles

There was a significant positive correlation between the Bray Curtis scores from the taxonomic analysis and the Bray Curtis scores from the functional gene analysis (*p* < 0.001, *r* = 0.271). This indicates that samples with similar taxonomic profiles tended to have similar functional gene profiles, and samples with dissimilar taxonomic profiles tended to have dissimilar functional gene profiles. In addition, the PCOA ordinations (Figures 4A, B) indicated that there was greater variation in taxonomic profiles across the samples (i.e., higher dissimilarity) than in functional gene profiles (i.e., less dissimilarity). This observation was confirmed by the fact that the average Bray Curtis dissimilarity score for the taxonomic profiles (0.605 ± 0.143) was higher than for the functional gene profiles (0.188 ± 0.068; ANOVA, *p* < 0.001). These results indicate that the bacterial assemblages from the different sampling sites and times varied more in taxonomy than in functional genes.

## 4 Discussion

Effluent from the SMP wastewater treatment plant was a point source of FPM and FPOM to La Tordera River, resulting in increases in both FPM and FPOM immediately downstream of the WWTP. A similar result was previously reported at this same site (Drummond et al., 2022). The nutrient content (%C, %N, %OM) of the FPM in the stream was also highest immediately downstream of the WWTP, indicating that the WWTP was a point source of high nutrient content fine particulates to the stream. The input of these high nutrient fine particles was correlated with an increase in microbial metabolic activity immediately downstream of the WWTP, which supports previous studies reporting that fine particles in streams are sites of high microbial activity (Ruggiero et al., 2006) and that WWTP effluent can result in increased rates of microbial metabolic activity (Burdon et al., 2020). This increase in microbial metabolic activity immediately downstream of the WWTP was accompanied by a significant decrease in the taxonomic richness and diversity of the bacterial assemblages associated with the fine particles as well as changes in their taxonomic and functional gene profiles. These results suggest either strong selective pressure on native communities caused by the effluent, or the input of fine particles from the effluent carrying bacterial assemblages of different composition and lower diversity than those sourced from upstream. The identification of 3,732 downstream-only OTUs which cumulatively accounted for 25% of the total sequences found at the downstream site demonstrated that the effluent was a significant source of particle-associated bacteria, although selection pressure may also have contributed to the composition of the downstream communities. Our study is the first to report the effects of WWTP effluent on the diversity and taxonomic composition of bacterial communities associated with fine particles, although decreases in bacterial richness and diversity and changes in taxonomic composition were previously observed for benthic biofilm communities downstream of the WWTP at this same field site (Freixa et al., 2020). Similar trends have also been reported for benthic communities at another site in Spain (Marti and Balcázar, 2014), at several sites in the Chicago metropolitan region (Drury et al., 2013; Lorentz et al., 2025), and at another site in Illinois (Hladilek et al., 2016), as well as for organic matter associated communities from 20 sites across Switzerland (Burdon et al., 2020) and for bacterioplankton communities in an intermittent stream in Catalonia, Spain (Pascual-Benito et al., 2020).

The significant correlation between the taxonomic and functional gene profiles of the particle-associated bacterial communities is another novel contribution of our study which suggests that similar environmental factors drove changes in both aspects of these communities. Moreover, the higher variation in taxonomic composition across sites compared to the lower variation in functional gene composition illustrates the high degree of functional redundancy within microbial communities and suggests a mechanism by which communities can maintain functionality despite changes in taxonomy, as demonstrated by the high rate of particle-associated microbial metabolic activity immediately downstream of the WWTP.

In our study the effects of effluent were short-lived and decreased with distance from the effluent input. Specifically, FPM concentration and nutrient content decreased, metabolic activity decreased, and bacterial richness and diversity increased at the sites farther downstream. In addition, bacterial assemblage taxonomic and functional gene composition at the sites farther downstream from the effluent input were more similar to the upstream assemblages, suggesting that bacterial assemblages were shifting toward a more native composition at sites farther from the effluent input. Similar recovery of bacterial taxonomic composition with distance from effluent input was previously reported for benthic biofilm communities at this same field site (Freixa et al., 2020), at sites below a WWTP in Charleston, IL (Hladilek et al., 2016), and for bacterioplankton communities in another intermittent stream in Spain (Pascual-Benito et al., 2020). Yet, our study is the first to report this type of recovery in FPM associated bacterial assemblages and to correlate it with recovery in functional gene profiles, which is especially important because a large fraction of these fine particles, sourced by the effluent, can travel long distances from the origin. Thus, the recovery of the natural communities suggests that the resilience of microbial biota associated with FPM can help to ameliorate the effects of WWTP effluents on downstream ecosystems and maintain ecosystem function.

Some of the most abundant downstream-only OTUs were assigned to the taxa Verrucomicrobia, Sphingobacteriales, and Bacteroidetes (Supplementary Table S1). Although Verrucomicrobia are generally a minor component of aquatic bacterial communities (Balmonte et al., 2016), they have been identified in WWTPs (Tyagi et al., 2025) and in sediments receiving WWTP effluent (Hou et al., 2025). Verrucomicrobia are known for their ability to metabolize a wide range of polysaccharides (Fuerst, 2019), so their association with FPM downstream may contribute to the decomposition of these particles. Sphingobacteriales is an order of Gram-negative bacteria known for their ability to metabolize anthropogenic compounds, including herbicides and antimicrobial compounds (Kämpfer, 2010). An increase in Sphingobacteriales sequences was also observed in sediment samples at several sites below WWTPs in the Chicago metropolitan region (Drury et al., 2013; Lorentz et al., 2025) and at a site below a WWTP in Charleston, IL (Hladilek et al., 2016). The abundance of Sphingobacteriales at sites below effluent inputs may reflect their ability to metabolize anthropogenic compounds typically found in wastewater (Bartelt-Hunt et al., 2009). Finally, Bacteroidetes is a phylum of Gram-negative bacteria that is common in stream biofilms (Battin et al., 2016) and in WWTPs (Tyagi et al., 2025) and includes taxa known for their resistance to antibiotics (Krieg et al., 2010), so their detection downstream may reflect the presence of antibiotics in the wastewater system.

Several taxa differed significantly in relative abundance in the near effluent samples as compared to the samples farther downstream, indicating a decrease with distance downstream. For example, at both the family level and OTU level sequences from the Rhodocyclaceae and Xanthomonadaceae families were significantly more abundant immediately downstream of the effluent input compared to either the upstream or the farthest downstream sites. This pattern suggests that the WWTP effluent input had a positive effect on these two families. Previous work reported that Rhodocyclaceae were abundant in stream sediments at sites receiving WWTP effluent (Hladilek et al., 2016; Martínez-Santos et al., 2018), in a wastewater impacted urban river (Newton and McLellan, 2015), and on organic substrates incubated in an urban river contaminated with sewage (Rosi et al., 2018). Similarly, Xanthomonadaceae were previously found to be more abundant in stream sediments downstream of WWTP inputs as compared to upstream at a site in northeast Ohio (Roberto et al., 2019). Rhodocyclaceae and Xanthomonadaceae are both families of Gram-negative bacteria that are common in WWTPs (Zhang et al., 2012; Wang et al., 2012), so their high abundance at sites downstream of WWTPs may reflect a direct input of these organisms via the effluent. Members of both the Rhodocyclaceae and Xanthomonadaceae families have also been linked to the removal of anthropogenic compounds in the environment or in biotechnological systems (Loy et al., 2005; Jayamani and Cupples, 2015; Li et al., 2018), so their high abundance in the FPOM of La Tordera River immediately downstream of the SMP WWTP may contribute to the breakdown of anthropogenic contaminants potentially present in the effluent. Finally, two Pseudomonas OTUs were more abundant immediately downstream of the WWTP than upstream, and their abundance continued to increase with distance from the WWTP. These data indicate that the WWTP was a point source of these taxa and that the environmental conditions within the stream favored their growth and colonization of FPM. This finding is not surprising considering that organisms from the Pseudomonas genus are common in freshwater ecosystems and are known for their ability to produce biofilms (Palleroni, 2010) and to degrade a large variety of organic molecules (Palleroni, 2010), making them important contributors to stream ecosystem function.

In contrast to the taxa mentioned above, other taxa were significantly less abundant in the near effluent samples and more abundant at the sites farther from the effluent input, suggesting a negative effect of the WWTP effluent on these taxa. For example, at both the family level and OTU level sequences from the Comamonadaceae family were most abundant in the upstream samples, were significantly less abundant immediately downstream of the effluent input and increased in abundance at the far from effluent sites. A decrease in Comamonadaceae sequences was also observed at several sites below WWTPs in the Chicago metropolitan region (Drury et al., 2013; Lorentz et al., 2025) and at a site below a WWTP in Charleston, IL (Hladilek et al., 2016). Previous work also reported that the abundance of Comamonadaceae on organic substrates incubated in several urban rivers was negatively affected by antibiotic exposure (Rosi et al., 2018), suggesting that the negative effect of effluent on Comamonadaceae that was observed in this study may be linked to anthropogenic contaminants common in wastewater, including pharmaceuticals and antimicrobial compounds that can be present in WWTP effluent (Bartelt-Hunt et al., 2009). Comamonadaceae is a family of Gram-negative aerobic heterotrophs that degrade a wide range of complex organic compounds in aquatic habitats (Garrity et al., 2005), so the negative effect of WWTP effluent on this group may be ecologically significant.

There were significant differences in the relative abundance of several groups of functional genes between the upstream site and the downstream sites, suggesting an impact of the effluent. The downstream sites had a higher abundance of genes involved in denitrification, which may have been driven by the lower level of dissolved oxygen at the downstream sites which would favor anaerobic metabolism. The potential impact of dissolved oxygen on the bacterial communities is supported by the negative correlation that was observed between dissolved oxygen and the taxonomic and functional gene profiles. Denitrification is a critical step in the nitrogen cycle that removes nitrogen from aquatic ecosystems and can reduce nutrient loading from WWTP effluent and limit eutrophication, so the increase in these functional genes may be ecologically significant. The 100 m downstream site also had a significantly higher abundance of genes encoding multidrug efflux pumps as compared to the upstream site. Multidrug efflux pumps are membrane transporters that can confer resistance to a wide range of stressors, including antibiotics, disinfectants, detergents, heavy metals, and organic solvents (Blanco et al., 2016), and bacteria expressing multidrug efflux pumps have been isolated from wastewater treatment systems (Sanderson et al., 2020) suggesting that the presence of some of these stressors in the wastewater may have selected for taxa with resistance to these stressors. The abundance of these genes within the particle-associated bacterial assemblages did not decrease significantly with distance, raising concerns about the dissemination of these resistant taxa in the environment. However, the lack of effluent effect on the abundance of genes conferring resistance to specific antibiotics suggests that antibiotics may not have been a significant driver of the effluent effects.

The results of the current study suggest that upstream flow, which was present in November but not in July, can moderate the effects of WWTP effluent on FPM accumulation, microbial activity, and bacterial assemblage composition in the receiving stream. At the site immediately downstream of the WWTP (100 m), FPM and FPOM were both lower in November than July, while taxonomic richness and Shannon diversity of bacterial assemblages were higher in November than July. In addition, microbial metabolic activity was consistently lower in November than July at all the downstream sites. These results suggest that the effect of effluent was greater in July, when there was no upstream flow. In addition, higher water temperature in July may have contributed to some of these trends, as higher temperature can increase microbial metabolic activity, and a recent study at another intermittent stream in Spain reported a significant negative correlation between temperature and bacterioplankton diversity (Pascual-Benito et al., 2020). Some bacterial taxa were significantly more abundant in the July downstream samples, including the genus *Aeromonas*, most species of which have been associated with human diseases, including gastrointestinal infections (Parker and Shaw, 2011). *Aeromonas* was 7-fold more abundant in July than November, representing almost 2% of total sequences in July samples, and was very rare in upstream samples, indicating that effluent was a point source of this organism, and that this organism was much more prevalent in the absence of upstream flow. Higher water temperature in July may also have favored the persistence of human gut-associated microbes like *Aeromonas*.

In contrast, several other bacterial taxa, specifically, Pseudomonadaceae, Comamonadaceae, and Flavobacteriaceae, were significantly more abundant in the November downstream samples as compared to the July downstream samples, and both Comamonadaceae and Flavobacteriaceae were also highly abundant in the November upstream samples, suggesting that the upstream flow served as a source of these taxa to the downstream sites. All three of these families are common stream inhabitants that are important contributors to organic matter cycling in streams, so the negative effect of effluent on these taxa may impact stream carbon cycling. The lower abundance of these taxa in the period without upstream flow suggests that the negative effect of effluent on these taxa may be most significant during low flow or in intermittent streams, which are predicted to become more prevalent due to climate change (Döll and Schmied, 2012). These results suggest that WWTP effluent can be a point source of microbes to the receiving stream and that dilution of WWTP effluent provided by natural stream flow can help promote common stream microbes and limit the abundance of wastewater-associated and potentially pathogenic microbes. This suggests that we should be concerned about the potential decreases in river flows and increases in the number of intermittent streams in many parts of the world that are likely to result from climate change. Care should be taken in extrapolating the results of this study too broadly since it was based on one field site and assessed the impacts of one WWTP at two time points. Future studies should consider applying this approach to a broader range of field sites and time points.

## Data Availability

Raw amplicon sequence data from this study have been posted to the National Center for Biotechnology Information (NCBI) Sequence Read Archive (SRA) with accession number PRJNA1451225. Metagenomic sequence data are available at the European Nucleotide Archive (ENA) with accession number PRJEB1112300.
