# Supplementary material for: Wastewater treatment plant effluent alters particle-associated bacterial assemblages in an intermittent stream
Source: Front Microbiol. 2026 Aug 11;17:1880555. doi: 10.3389/fmicb.2026.1880555 (PMC13503315; doi:10.3389/fmicb.2026.1880555)
Supplement: Supplementary file 1 [file Data_Sheet_1.pdf]

Wastewater Treatment Plant Effluent Alters Particle-Associated Bacterial Assemblages in an Intermittent Stream

Supplemental Material

John J. Kelly[1,*], Karl Gaisser[1], Jennifer D. Drummond[2], José Gonçalves[3], Susana Bernal[3], and Eugènia Martí[3]

[1] Loyola University Chicago, Chicago, IL, USA
[2] University of Birmingham, School of Geography, Earth and Environmental Science, Birmingham, UK
[3] MARE – Marine and Environmental Sciences Centre, ARNET – Aquatic Research Network, NOVA School of Science and Technology, NOVA University Lisbon, Campusda Caparica, 2829-516, Caparica, Portugal
[4] Integrative Freshwater Ecology Group, Centre of Advanced Studies of Blanes (CEAB-CSIC), Blanes, Spain

***** Correspondence:
John J. Kelly
Department of Biology
Loyola University Chicago
1032 West Sheridan Rd.
Chicago, IL 60660
phone: 773.508.3681
email: jkelly7@luc.edu

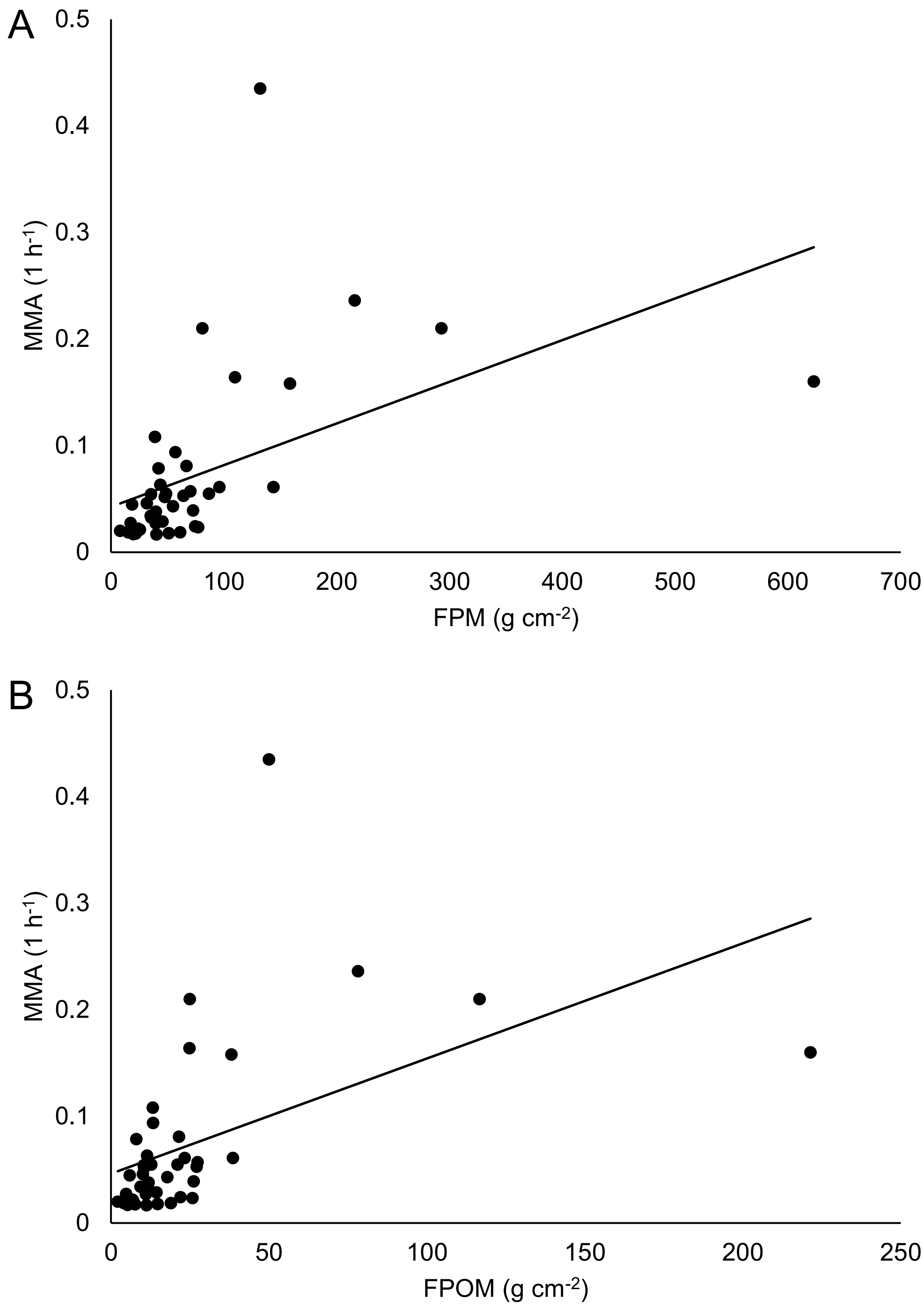

Figure S1. Relationships between (A) MMA and FPM (r=0.493, p=0.001) and (B) MMA and FPOM (r=0.497, p=0.001).

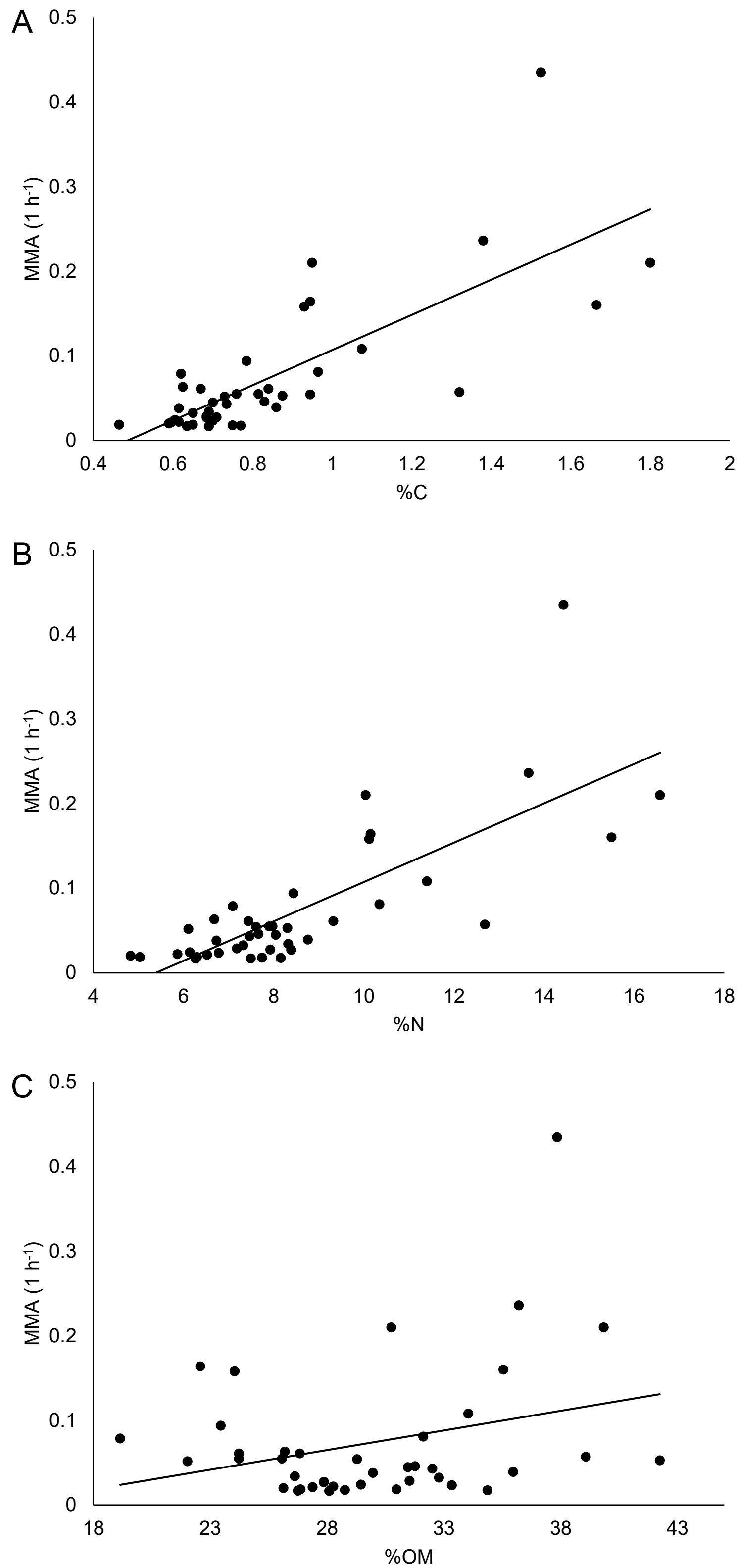

Figure S2. Relationships between (A) MMA and %C (r=0.758, p<0.001), (B) MMA and %N (r=0.769, p<0.001), and (C) MMA and %OM (r=0.293, p=0.063).

Table S1. Most abundant bacterial OTUs found in the 100 m downstream samples but absent from the upstream samples

OTU [a]	Percentage of Total Sequences [b]
Verrucomicrobia Subdivision 3	1.65%
Gammaproteobacteria	0.65%
Sphingobacteriales	0.55%
Bacteria unclassified	0.36%
Bacteroidetes	0.34%
Arcobacter	0.30%
Bacteroidetes	0.29%
Acidobacteria Group 10	0.29%
Vampirovibrio	0.28%
Cryomorphaceae	0.27%
Bacteroidetes	0.25%
Bacteroidetes	0.25%
Bacteria unclassified	0.24%
Bacteria unclassified	0.24%
Flavobacteriales	0.24%
Burkholderiales	0.23%
Sphingobacteriales	0.21%
Sphingobacteriales	0.20%
Chitinophagaceae	0.20%
Azospira	0.17%
Bacteroidetes	0.16%
Bacteria unclassified	0.14%
Bacteroidetes	0.14%
Cystobacteraceae	0.13%
Flavobacteriales	0.13%
Rhodocyclaceae	0.13%
Bacteroidetes	0.12%
Deltaproteobacteria	0.12%
Bacteroidetes	0.12%
Caldilinea	0.12%
Bacteroidetes	0.12%
Actinomycetales	0.11%
Betaproteobacteria	0.11%
Thauera	0.11%
Cloacibacterium	0.11%
Acidobacteria Group 3	0.10%
Betaproteobacteria	0.10%
Fluviicola	0.10%
Bacteroidetes	0.10%

[a] OTUs were identified to the lowest possible taxonomic rank and arranged in order of relative abundance. Only OTUs accounting for greater than 0.1% of total sequences were included in the table.

[b] Percentage of total sequences in the 100 m downstream samples.

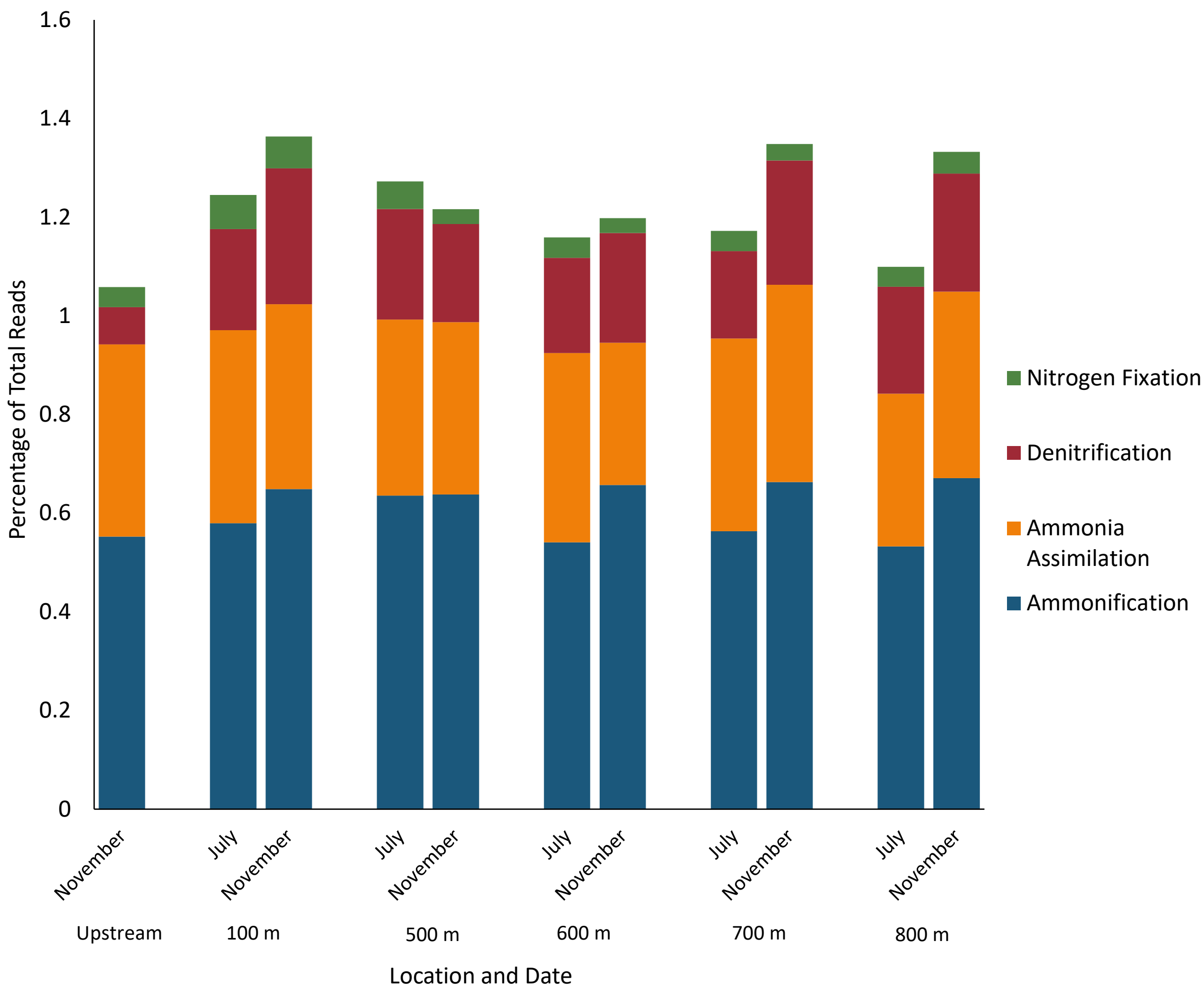

Figure S3. Spatial patterns of average relative abundance of functional genes related to nitrogen metabolism within bacterial assemblages associated with fine particular matter upstream and at several distances downstream of the wastewater treatment plant effluent input. Data represent mean values (n=3 for downstream sites and n=6 for upstream).

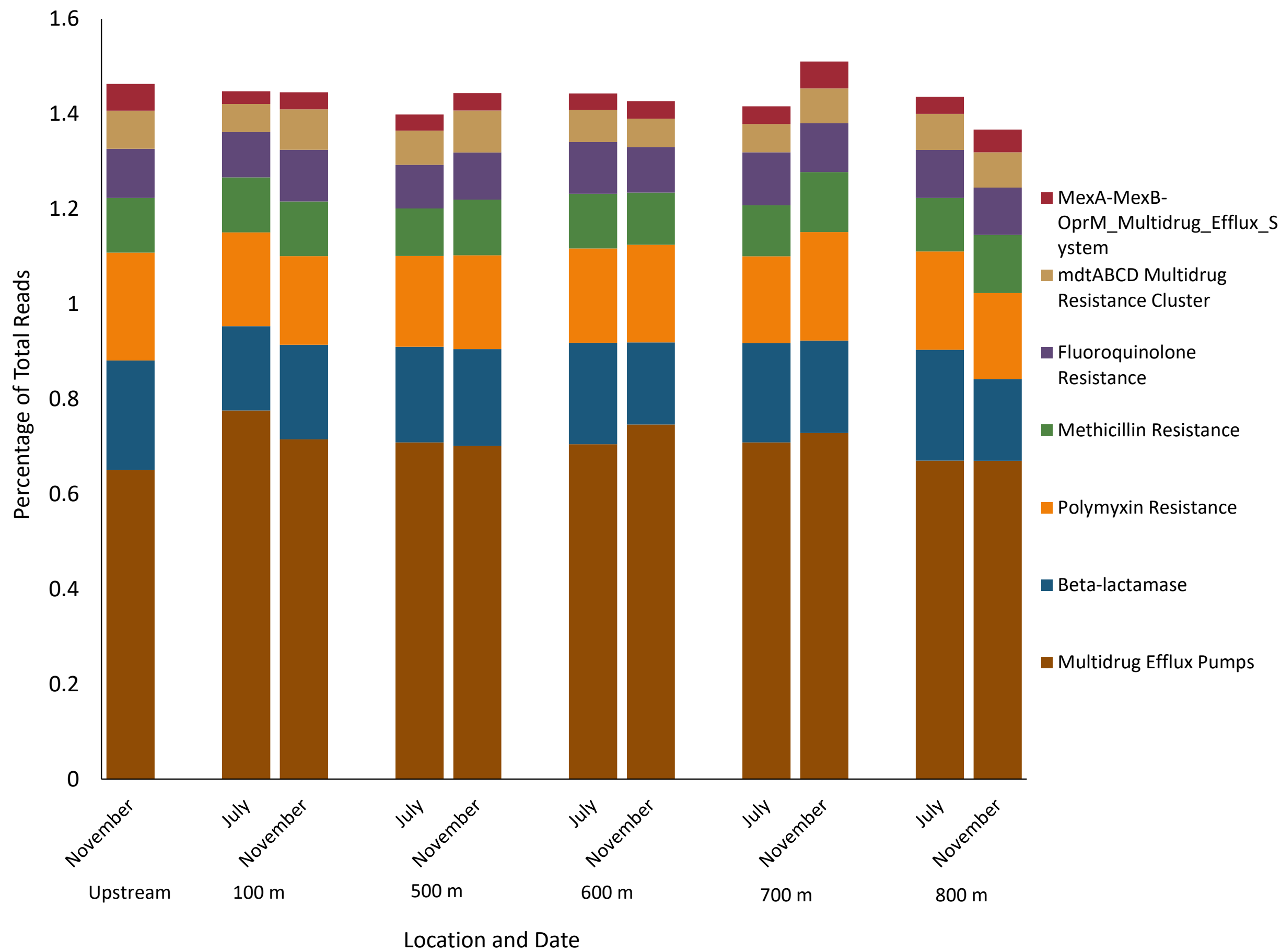

Figure S4. Spatial patterns of average relative abundance of functional genes related to antibiotic resistance within bacterial assemblages associated with fine particular matter upstream and at several distances downstream of the wastewater treatment plant effluent input. Data represent mean values (n=3 for downstream sites and n=6 for upstream).

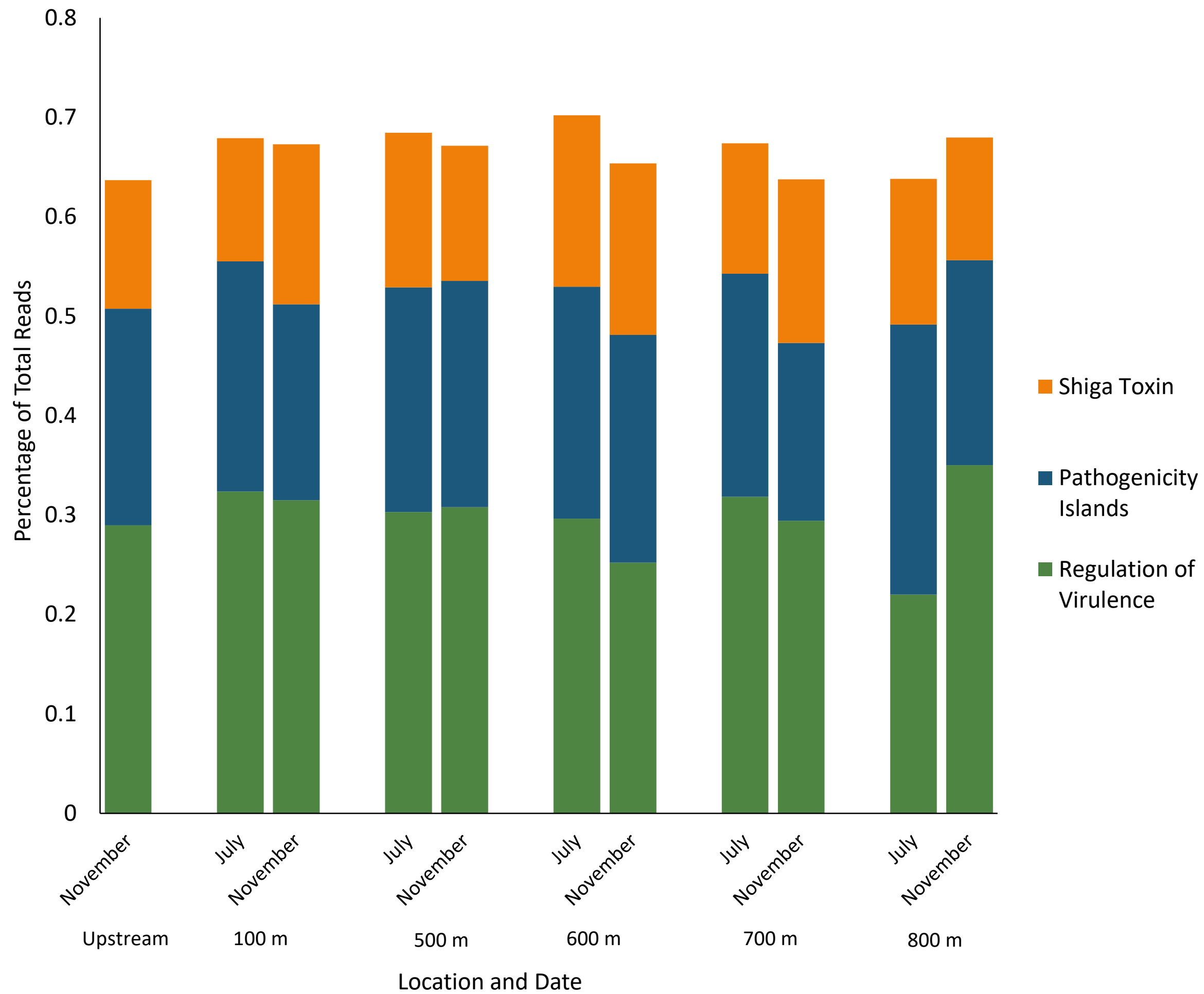

Figure S5. Spatial patterns of average relative abundance of functional genes related to virulence within bacterial assemblages associated with fine particular matter upstream and at several distances downstream of the wastewater treatment plant effluent input. Data represent mean values (n=3 for downstream sites and n=6 for upstream).
